# Large and small herbivores have strong effects on tundra vegetation in Scandinavia and Alaska

**DOI:** 10.1002/ece3.7977

**Published:** 2021-08-02

**Authors:** Elin Lindén, Laura Gough, Johan Olofsson

**Affiliations:** ^1^ Department of Ecology and Environmental Science Umeå University Umeå Sweden; ^2^ Department of Biological Sciences Towson University Towson Maryland USA

**Keywords:** Arctic, diversity, exclosures, Herbivores, plant communities

## Abstract

1. Large and small mammalian herbivores are present in most vegetated areas in the Arctic and often have large impacts on plant community composition and ecosystem functioning. The relative importance of different herbivores and especially how their specific impact on the vegetation varies across the Arctic is however poorly understood.
2. Here, we investigate how large and small herbivores influence vegetation density and plant community composition in four arctic vegetation types in Scandinavia and Alaska. We used a unique set of exclosures, excluding only large (reindeer and muskoxen) or all mammalian herbivores (also voles and lemmings) for at least 20 years.
3. We found that mammalian herbivores in general decreased leaf area index, NDVI, and abundance of vascular plants in all four locations, even though the strength of the effect and which herbivore type caused these effects differed across locations. In three locations, herbivore presence caused contrasting plant communities, but not in the location with lowest productivity. Large herbivores had a negative effect on plant height, whereas small mammalian herbivores increased species diversity by decreasing dominance of the initially dominating plant species. Above‐ or belowground disturbances caused by herbivores were found to play an important role in shaping the vegetation in all locations.
4. *Synthesis:* Based on these results, we conclude that both small and large mammalian herbivores influence vegetation in Scandinavia and Alaska in a similar way, some of which can mitigate effects of climate change. We also see important differences across locations, but these depend rather on local herbivore and plant community composition than large biogeographical differences among continents.

## Graphical abstract

Large and small herbivores have strong effects on plant community structure in both Alaska and Scandinavia. The effects are more dependent on herbivore type and site productivity than biogeographical differences between regions.

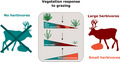

## 1 INTRODUCTION

Mammalian herbivores are key drivers of plant community composition and ecosystem functioning in the Arctic (Jefferies et al., 1994; Olofsson & Post, 2018). Although the species richness of herbivores is low, large mammalian ungulates and microtine rodents are present in most vegetated arctic areas (Barrio et al., 2016; Olofsson & Post, 2018). The vast majority of species are small rodents like lemmings and voles (Ehrich et al., 2020), and only two large herbivore species have a wider spatial distribution, reindeer/caribou (*Rangifer tarandus*) (Uboni et al., 2016), and muskoxen (*Ovibos moschatus*) (Cuyler et al., 2020). With a herbivore diversity this low, the presence of several herbivore types is likely to have strong effects on arctic vegetation, since a more diverse herbivore guild could target a more diverse assembly of plant species (Olofsson & Post, 2018).

Large herbivores affect plant community structure and ecosystem functions in various ways. At local scales, they typically promote grazing tolerant graminoids at the expense of trampling sensitive mosses and lichens (Gough et al., 2008; van der Wal, 2006). Large herbivores also hold back shrub vegetation, which gives them potential to mediate some effects of climate change on tundra plant communities (Bråthen et al., 2017; Christie et al., 2015; Manseau et al., 1996). They also increase species diversity and rare plant species occurrence on both local and regional scale (Kaarlejärvi et al., 2017; Olofsson & Oksanen, 2005; Sundqvist et al., 2019). Often, this comes from selective feeding or physical disturbance on dominant and grazing intolerant plant species, which creates gaps in the vegetation where new species can establish (Kaarlejärvi et al., 2017). Also, belowground processes, such as nutrient turnover (Barthelemy et al., 2017), carbon fluxes (Metcalfe & Olofsson, 2015), and carbon sequestration (Väisänen et al., 2014), are partly controlled by large herbivores. Although the strong effects of herbivores found at local scales are difficult to generalize for larger areas (Bernes et al., 2015), a regional study across the fennoscandian mountain chain showed that reindeer reduced the abundance of lichens, deciduous shrubs, and plant density (NDVI and LAI) and increased soil nutrient availability (Sundqvist et al., 2019). Further, the effect of reindeer on species richness shifted from negative to positive across a gradient of increasing productivity (Sundqvist et al., 2019).

Microtine rodents, voles and lemmings being most common, are also key species in arctic ecosystems, since they influence plant community composition and serve as food for predators (Legagneux et al., 2012). They are well known for their multiyear cyclic population dynamics with alternating high (outbreaks) and low densities throughout the Arctic (Ehrich et al., 2020). During population peaks, their impact on the vegetation is often stronger than the effect of large herbivores (Olofsson et al., 2004; Petit Bon et al., 2020) and sometimes so strong that it can be detected from satellite images (Olofsson et al., 2012). Compared to large herbivores, their presence leads to a more pronounced decrease in dwarf shrubs and higher shrubs (Olofsson et al., 2009), as well as moss and lichen biomass (Johnson et al., 2011; Moen et al., 1993). Even though they are more sensitive to deterrent plant secondary metabolites (Batzli & Jung, 1980) than large herbivores, voles and lemmings can still reduce biomass of less palatable plants substantially through physical disturbance from runways (Olofsson et al., 2004, 2012).

Despite their circumpolar distribution and potential to influence circumpolar phenomena like the greening of the Arctic (Myers‐Smith et al., 2011), little is known about whether and how the ecological importance of large and small herbivores varies across Arctic ecosystems. Since most parts of the Arctic are inhabited by only one species of large herbivore, predominantly reindeer (Olofsson & Post, 2018), and/or small herbivores of similar ecological function (Ehrich et al., 2020), effects on the vegetation could be expected to be similar. However, there are also good reasons to believe that the effect of herbivores might differ across the Arctic, given that herbivore density, guild composition, and management (domestication and hunting) differ among regions (Olofsson & Post, 2018), and so does plant community structure (Myers‐Smith et al., 2020). Large‐scale depletion of vegetation is observed during peak rodent years in Scandinavia (Hoset et al., 2014; Olofsson et al., 2012), and it has been argued that these strong effects are not occurring everywhere in the Arctic (Gauthier et al., 2009). Still, no studies have directly tested whether the strength of the interactions between small rodents and vegetation varies across the Arctic. It has also been proposed that the strong effects of reindeer on vegetation are primarily linked to domesticated reindeer, but a lack of studies in areas with wild reindeer/caribou prevent solid tests of such hypothesis (Bernes et al., 2015). A more detailed understanding of how the role of different types of herbivores varies across the Arctic is needed to facilitate future predictions on how these ecosystems will change in a warming future.

A first step in investigating the importance of large and small herbivores across the Arctic is to compare results from experiments conducted across the Arctic using comparable data. We here compare the long‐term impact of large and small mammalian herbivores on the vegetation in four locations of differing vegetation type, two in Alaska and two in Scandinavia. All four locations have very similar experimental setups with large and small mesh size exclosures established between 1989 and 1998. We recorded plant density and composition using the same methods across all locations in the same year. Although these four locations are not representative of the Arctic as a whole (Metcalfe et al., 2018), comparing these locations is a first attempt to experimentally study how the effect of small and large mammalian herbivores varies across the Arctic. Based on previous findings, we hypothesize that:

1. Herbivores change vegetation structure and composition in similar ways in both Scandinavian and Alaskan tundra, but the effects differ among vegetation types.
2. Small mammalian herbivores have a larger effect on vegetation than large herbivores.
3. The presence of all mammalian herbivores leads to higher diversity of tundra vegetation.

## 2 MATERIALS AND METHODS

### 2.1 Study area and experimental design

**FIGURE 1:**
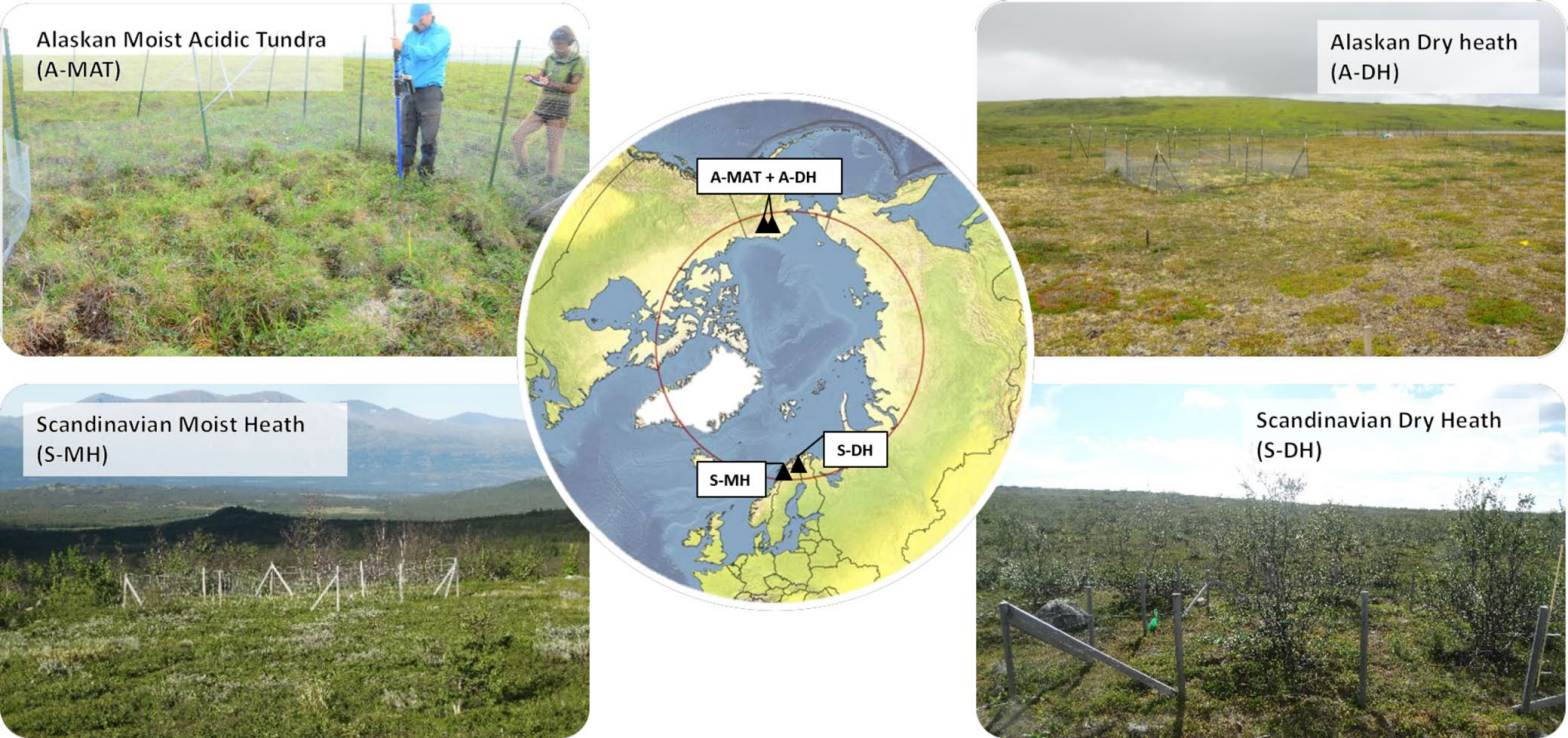
Photographs showing experimental exclosure setups used to study the effect of large and small herbivores on tundra vegetation in four tundra locations. Two in Alaska (A‐MAT = Alaskan moist acidic tundra, A‐DH = Alaskan dry heath) and two in Scandinavia (S‐MH = Scandinavian moist heath, S‐DH = Scandinavian dry heath). At each location, there are three levels of exlosure treatments allowing no mammal herbivores, only small mammal herbivores or all mammalian herbivores to access the vegetation

We compared the effect of small and large herbivores on tundra vegetation between four locations, two in Scandinavia and two in Alaska (Figure 1). In all four locations, there are three types of exclosure treatments to exclude or permit access of small and large herbivores. The naming of the three treatments was based on the size class of herbivores with access to the vegetation: Fine mesh sized wire fence excluded both small (hares and microtine rodents) and large (reindeer/caribou, muskoxen) mammalian herbivores (no herbivores, NH) and were considered ungrazed controls. Large mesh sized fences kept out large herbivores but allowed small herbivores access the vegetation (small herbivores, SH). Both small and large herbivores could access freely grazed, open plots (all herbivores, AH). All exclosures were built between 1989 and 1998 and are thus between 20 and 30 years old (Table 1).

**TABLE 1:** Information on the experimental setup in four tundra locations in Scandinavia and Alaska (S‐MH = Scandinavian Moist Heath, S‐DH = Scandinavian Dry Heath, A‐MAT = Alaskan Moist Acidic Tundra, A‐DH = Alaskan Dry Heath) containing experimental plots with three levels of herbivore presence (NH = no herbivores, SH = small herbivores, AH = all herbivores)

Location	Established (year)	Experimental plot size (m)	Included blocks (available blocks on site)	Subplots per plot (NH + SH + AH)	Herbivores present
S‐MH	1998	8 × 8	3 (3)	8 + 8 + 8	Reindeer, lemming, vole
S‐DH	1998	8 × 8	3 (3)	8 + 8 + 8	Reindeer, lemming, vole
A‐MAT	1996	5 × 5	2 (4)^c^	8 + 8 + 8	Caribou, vole
	1989	10 × 10^a^ (SH + AH) 2.5 × 2.5 (NH)	2 (2)	8 + 8 + 4^b^	
A‐DH	1996	5 × 5	3 (3)^c^	8 + 8 + 8	Caribou, vole
	1989	10 × 10^a^ (SH + AH) 2.5 × 2.5 (NH)	2 (2)	8 + 8 + 4^b^	

^a^ Other treatments nested within SH, the area corresponding to SH is approximately 8.6 × 8.6 m. Also, due to lack of permanent open control plots AH subplots was placed along the outside of two sides of the SH treatment.

^b^ Only four plots could fit in the NH treatment.

^c^ Blocks excluded due to signs of nonintact exclosures.

Location 1 is situated close to Abisko, Sweden (68°19′23″N, 18°51′57″E). The mean annual temperature is 0.3°C, and the mean annual precipitation is 345 mm/year (Abisko Scientific Research Station, 4 km from the location; for more information on abiotic conditions in the sites during the year of inventories see Figure S1). The experimental plots are located in a productive dwarf shrub‐dominated heath (Scandinavian moist heath, S‐MH) where *Empetrum nigrum* ssp. *hermafroditum* is the most common species and the only taller shrub is *Betula nana* ssp. *nana*. Location 2 is situated close to Joatka, Norway, (69°45′11″N, 24°00′10″E). The mean annual temperature is −2.4°C, and the mean annual temperature is 443 mm/year (temperature from Suolovuopmi and Suolovuopmi‐Lulit stations at 380 m a.s.l., 25 km southwest of the study site, precipitation from Joatkajavre station (1999–2006) 1 km from the study sites). The experimental plots are located in lichen‐dominated dry heath vegetation (Scandinavian dry heath, S‐DH) with a field layer of mainly *Betula nana* ssp. *nana*, *Empetrum nigrum* ssp. *hermafroditum*, and *Vaccinium myrtillus*. In both locations, there are three blocks with three 8 × 8 m experimental plots (NH, SH, and AH), established in 1998. The SH plots consist of a 1.2‐m high large mesh size net excluding large herbivores but allowing small herbivores to pass, and the NH plots additionally have a 1‐m high small mesh size (1.2 × 1.2 cm) net dug down 10–30 cm into the mineral soil allowing no herbivores inside. Semidomesticated reindeer (*R. tarandus*) are the most abundant large herbivore in these locations, with moose (*Alces alces*) occasionally visiting the sites, and gray‐sided voles (*Myodes rufocanus*) and Norwegian lemmings (*Lemmus lemmus*) are the most abundant small herbivores (Olofsson et al., 2004).

Location 3 is situated close to Toolik Lake, Alaska (68°37′27″N, 149°36′36″E). The mean annual temperature is −8°C and the mean annual precipitation is 274 mm/year (Environmental Data Center Team, 2020). The dominant vegetation type is moist acidic tussock tundra (Alaskan moist acidic tundra, A‐MAT) with *Eriophorum vaginatum* as the dominant tussock building species, *Betula nana* ssp. *exilis* and *Rubus chamaemorus* as common elements, and a ground layer of *Sphagnum* mosses. Two 10 × 10 m large mesh size exclosures with nested 5 × 5 m medium mesh size exclosures, and 2.5 × 2.5 m small mesh size exclosures were built in 1989, and four paired 5 × 5 m large mesh size exclosures and 5 × 5 m small mesh size exclosures were built in 1996. A few exclosures had been invaded by voles or lemmings. To ensure that we studied the effect of completely excluding rodents, we excluded all blocks with obvious signs of small rodents (feces, burrows, runways) inside small mesh exclosures; two blocks from 1989 and two blocks from 1996 were sampled. We used only the small mesh exclosures (NH) and large mesh exclosures (SH) in the experiment from 1989; medium mesh exclosures were omitted since no comparable treatment existed in other blocks in Alaska or in Scandinavia. Permanent control plots were not available for the exclosures from 1989; for those, we placed our control subplots along two sides of each exclosure.

Location 4 is also situated close to Toolik Lake, Alaska (68°38′25″N, 149°35′15″E), and has the same climatic conditions as location 3. This location is located in a dry lichen heath habitat (Alaskan dry heath, A‐DH), with lichens dominating the ground layer, and the most common vascular plants are deciduous dwarf shrubs (*Arctostaphylos alpina*) and dwarf evergreen shrubs (*Empetrum nigrum* and *Loiseleuria procumbens*). Here, the exclosures have the same design and age as in location 3, with three blocks from 1996 and two blocks from 1989 which all were intact and included in our study. In locations 3 and 4, the most abundant large herbivore is caribou (*R. tarandus*); muskoxen also occur in the area but have not been observed at the experimental site. The most abundant microtine rodents are the tundra vole (*Microtus oeconomus*) and singing vole (*Microtus miurus*), but brown lemmings (*Lemmus trimucronatus*) and Arctic ground squirrel (*Spermophilus parryi*) are also present in the area (Batzli & Lesieutre, 1995). Locations 3 and 4 are both a part of the Arctic Long‐Term Ecological Research (LTER) project (https://arc‐lter.ecosystems.mbl.edu/).

### 2.2 Vegetation analyses

We recorded plant community composition and species abundance during July and August 2018 in eight subplots (50 × 50 cm) within each experimental plot (NH, SH, and AH), except for the NH plots from 1989 in locations 3 and 4, where we could only fit four subplots. To survey the plant community metrics, we used the point intercept method (Goodall, 1952) with 50 pins per subplot arranged in five 50 cm wide rows of ten vertical pins every 10 cm. We recorded the total number of hits for each separate vascular plant species on each pin, but only one contact per pin for ground hits (mosses, lichens, bare soil, etc.). In the two Alaskan locations, we also recorded standing dead *Eriophorum* litter. In each subplot, we measured the height of the tallest individual of deciduous shrub, evergreen shrub, graminoid, and forb, and the thickness of moss and lichen layer (one measure per subplot), with an accuracy of 0.5 cm.

For each subplot, we estimated vegetation density as leaf area index (LAI), normalized difference of vegetation index (NDVI), and total number of plant hits during pin‐pointing (see above). LAI (m^−2^ m^−2^) was estimated nondestructively using an AccuPAR LP‐80, Decagon devices (Wilhelm et al., 2000). We took three measurements below the vegetation and two above to encompass the spatial heterogeneity within subplots. We also measured NDVI from 2 m above each subplot using a hand‐held pole and 2 channel sensors (SKR1800D/SS2, SKL925 logger, SpectroSense2+, Skye instruments, Llandrindod Wells). The measurements at subplot level were used to calculate mean LAI and NDVI at plot level.

### 2.3 Data analyses

All point frequency data from the vegetation survey were standardized to number of hits per 100 pins (Väisänen et al., 2014) and used to represent plant abundance; relative abundance among species was further used to estimate plant community composition. Data from two subplots were incomplete and therefore omitted from all further analyses. For the remaining subplots, we calculated Simpson's diversity index using all species detected during the vegetation survey.

To investigate the effect of herbivores on plant community composition in our four locations, we performed separate NMDS analyses using Bray–Curtis dissimilarity matrix (“metaMDS” function in the “vegan” package (Oksanen et al., 2019)) for each location. We excluded rare species that only occurred in one plot (AH, SH, NH) in a location prior to the analyses. To test the main and interactive effect of treatment and site location on vegetation density, plant functional group abundance, vegetation height, and plant community diversity (Simpson's diversity index), we used linear mixed models with block (paired AH + SH + NH treatments) as a random factor (“lme” function in the “nlme” package, (Pinheiro et al., 2021)). If a significant treatment × location interaction indicated differences among locations, we carried out separate linear mixed models to test for treatment effects at each location. When no interactive effects were found, we simplified the models to test for main effects only and used parameter statistics as post hoc tests. For comparisons between treatment levels, we used “no herbivore” as reference level. All statistical analyses were performed in the statistical package R (R‐CoreTeam, 2021).

## 3 RESULTS

### 3.1 Effects of large and small herbivores on vegetation density

**FIGURE 2:**
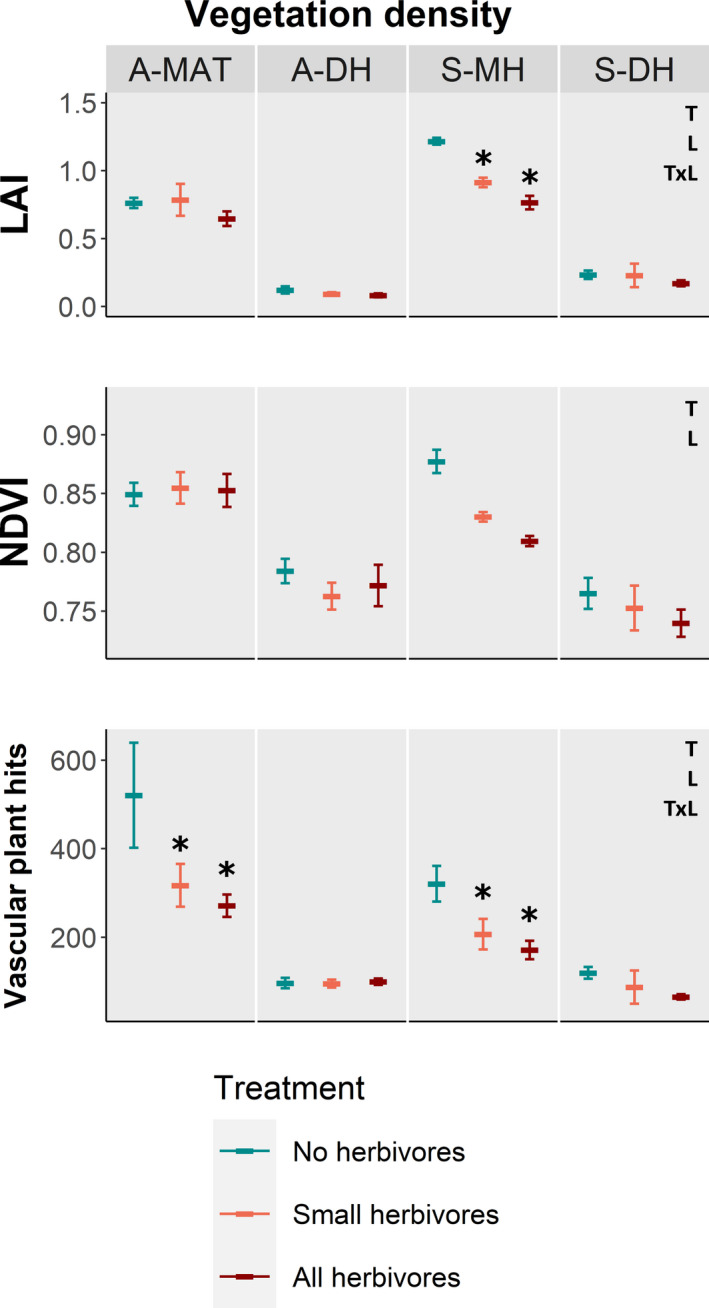
Effect of the presence of no herbivores (blue), small herbivores only (pink), and both small and large (all) mammal herbivores (red) on vegetation density in four tundra locations (A‐MAT = Alaskan moist acidic tundra, A‐DH = Scandinavian dry heath, S‐MH = Scandinavian moist heath, S‐DH = Scandinavian dry heath). Vegetation density is given as measured leaf area index (LAI), normalized difference of vegetation index (NDVI) and total field layer hits. Plotted values are means ± SE and letters on the figure's right side indicate significant explanatory variables from linear mixed models (T = treatment, L = location, TxL = interaction). For significant interactions, local treatment effects are marked out with asterisks

Vegetation density was lower with herbivores present, as indicated by LAI, NDVI, and total hits on vascular plants, compared to 2–3 decades of herbivore exclusion, but the effects differed among locations (Figure 2; Table 2) and measuring method (Figure 2). LAI was lower when both large and small herbivores had access to the vegetation, but the strength of the effect differed among locations (T × L, Table 2). In S‐MH, small herbivores caused a 0.31 units lower LAI and all herbivores further 0.15 units lower LAI (Figure 2) compared to when no herbivores could access the vegetation. In S‐DH, A‐DH, and A‐MAT, there were no statistically significant differences within locations (Figure 2).

**TABLE 2:** *F*‐values from linear mixed models analyzing the effect of small herbivores (microtine rodents) and copresence of small and large herbivores (also reindeer and muskoxen) (treatment; T) on LAI, NDVI, abundance of plant functional groups and standing dead plant material, vegetation height, and plant community diversity in four different locations (L; Scandinavian moist heath, Scandinavian dry heath, Alaskan moist acidic tundra, and Alaskan dry heath)

	*df*	Treatment (T)	Location (L)	T * L
		22	11	22
Vegetation density	LAI	12.40***	118.67***	5.39**
	NDVI	5.018**	22.45***	2.24
	Field layer hits	13.68**	13.90***	4.26***
Plant abundance	Deciduous shrubs	5.98**	6.91**	2.51
	Deciduous dwarf shrubs	3.06	7.71**	2.06
	Evergreen shrubs	7.81**	31.47***	6.34***
	Graminoids	6.20**	22.32***	6.68***
	Forbs	3.50*	11.30**	2.96*
	Mosses	1.26	74.15***	2.20
	Lichens	5.41*	44.98***	0.79
	Standing dead^a^	60.97***, *df* = 2	74.23***, *df* = 1	76.09***, *df* = 2
Vegetation height	*Betula nana* ssp.^b^	52.31***, *df* = 16	72.94***, *df* = 10	2.04, *df* ** = **16
	*Salix* sp.	NA	NA	NA
	Deciduous shrubs	40.25***	179.75***	6.21***
	Evergreen shrubs	10.40***	75.66***	2.52
	Graminoids	NA	NA	NA
	Forbs	NA	NA	NA
	Mosses^b^	3.98*, *df* = 18	30.85***, *df* = 11	0.66, *df* ** = **18
	Lichens	22.99***	10.98**	4.40**
Species diversity	Simpson's diversity index	3.88*	11.18**	0.43

Note

Statistical significance (*p < 0.05, **p < 0.01, ***p < 0.001) is written as boldface.

^a^ Model only contain Alaskan moist acidic tundra (A‐MAT) and Alaskan dry heath (A‐DH).

^b^ Model only contain plots where the growth form was present.

Across all locations, NDVI was on average 0.017 units lower with small herbivores present, and 0.021 lower when all herbivores were present compared to controls with no herbivores (T; Table 2). No statistical support for differences in strength of treatment effects among locations was found (T × L; Table 2), although it appeared to be stronger in the Scandinavian locations (Figure 2). Not surprisingly, NDVI was lower in dry heath locations than in locations with mesic heath and tussock tundra (Figure 2; L, Table 2). Point framing showed fewer vascular plant hits when herbivores had access to the vegetation in all locations except A‐DH (Figure 2), but the strength of the effects varied across sites (T × L; Table 2), and these outcomes were only statistically significant across treatments in the productive sites, S‐MH and A‐MAT (Figure 2).

### 3.2 Effects of large and small herbivores on plant community composition

**FIGURE 3:**
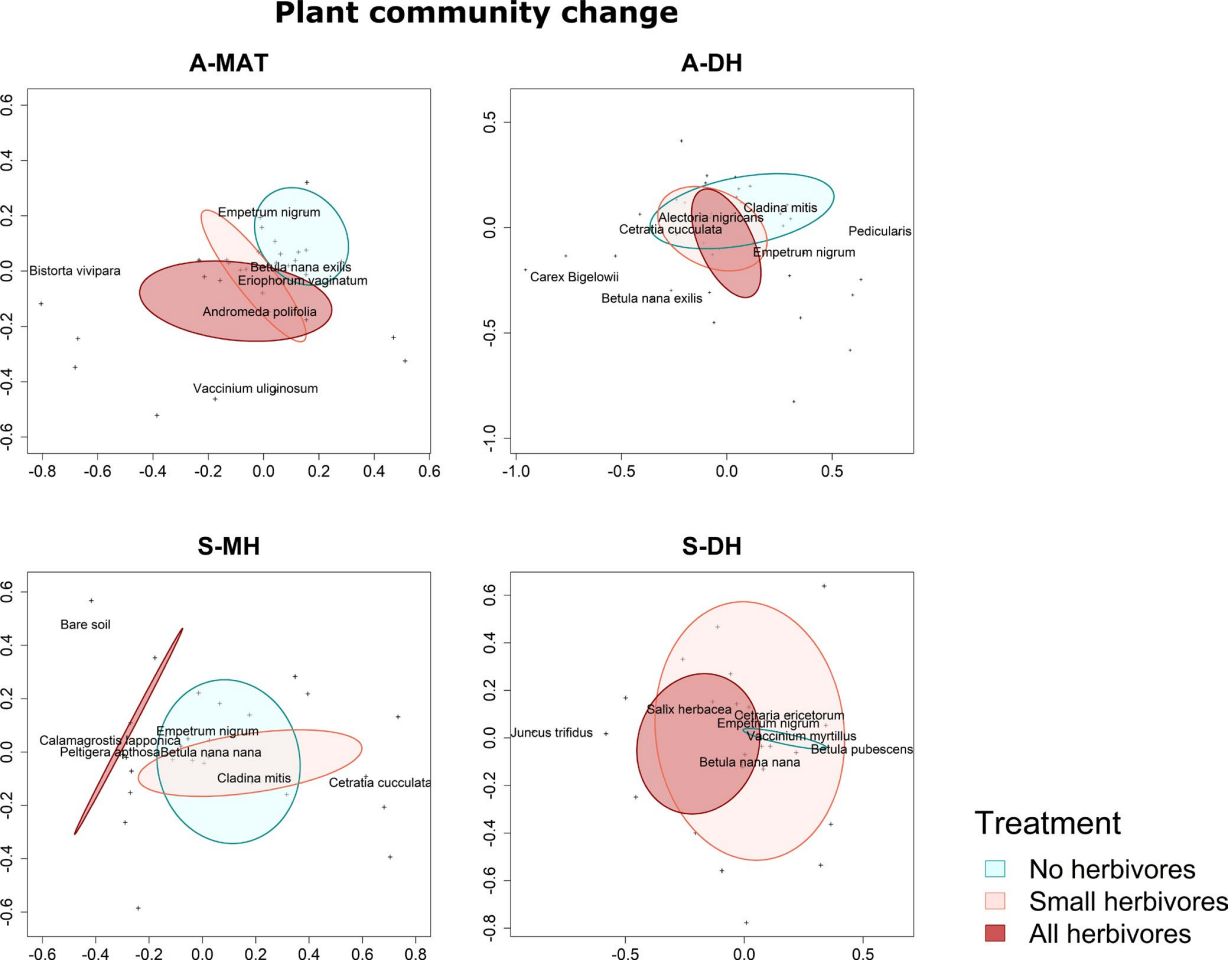
NMDS's showing differences in community composition among herbivory treatments in two Alaskan (A‐MAT = Alaskan moist acidic tundra, A‐DH = Alaskan dry heath) and two Scandinavian (S‐MH = Scandinavian moist heath, S‐DH = Scandinavian dry heath) tundra sites. Three levels of treatment (presence of mammalian herbivores) are indicated in the plots as: no herbivores (blue), small herbivores only (pink), and copresence of small and large (all) herbivores (red). The range of the ellipses represents the 95% confidence interval of standard error for treatment means

In three of the four locations, the long‐term absence of herbivores resulted in contrasting plant communities, as indicated by nonoverlapping ranges in the NMDS analyses, while we found no differences in plant community composition between the different herbivore regimes in A‐DH (Figure 3). The separate effects of small and large mammal herbivores differed among the other three locations (Figure 3). In A‐MAT, the vegetation difference between treatments was mostly driven by small herbivores, while in S‐MH and S‐DH, the additional presence of large herbivores had the strongest effect on plant community composition (Figure 3).

**FIGURE 4:**
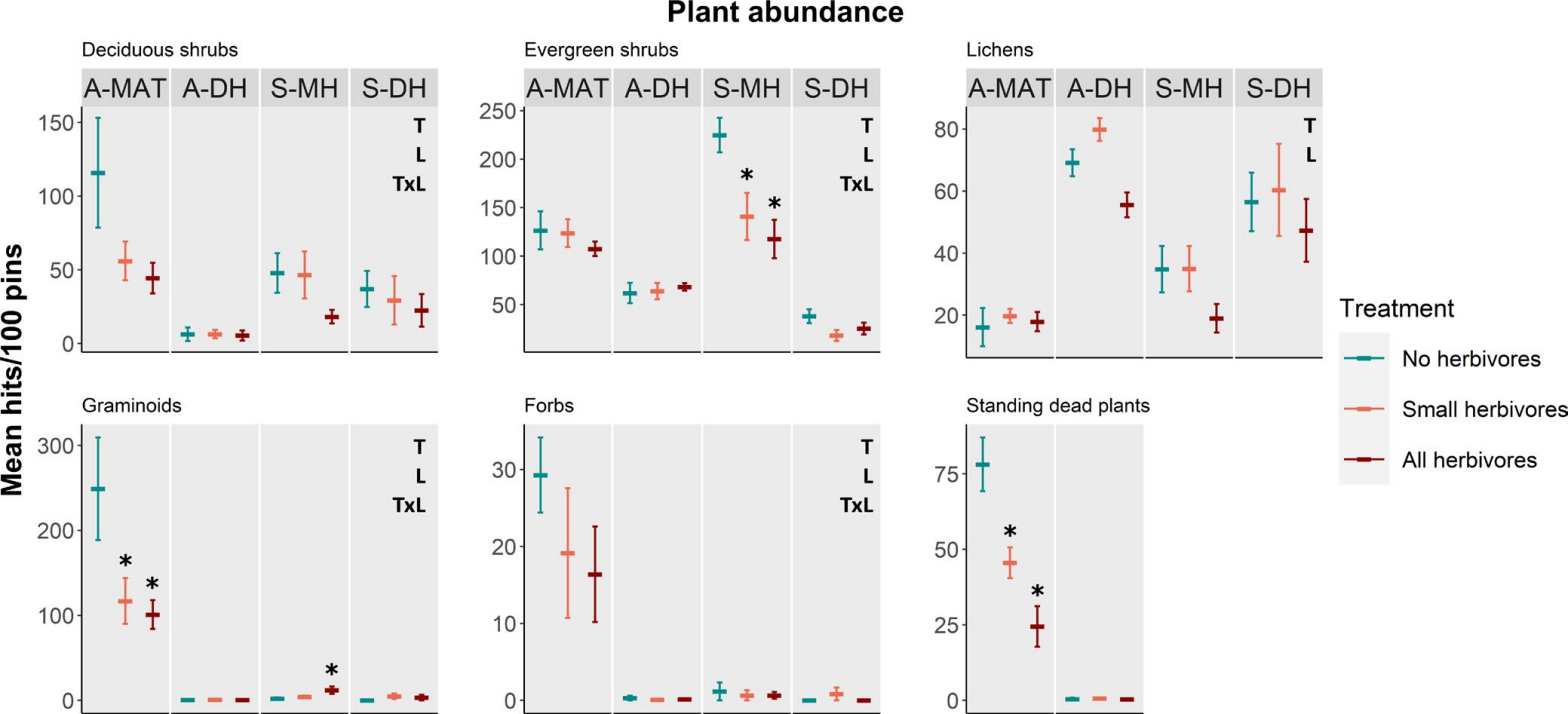
Effect of the presence of no herbivores (blue), small herbivores only (pink), and both small and large (all) mammal herbivores (red) on abundance of plant functional groups (tall‐growing deciduous shrubs, evergreen shrubs, lichens, graminoids, and forbs) in four tundra locations (A‐MAT = Alaskan moist acidic tundra, A‐DH = Alaskan dry heath, S‐MH = Scandinavian moist heath, S‐DH = Scandinavian dry heath), and abundance of standing dead plant material in two Alaskan tundra locations. Plotted values are means ± SE and letters on the figure's right side indicate significant explanatory variables from linear mixed models (T = treatment, L = location, TxL = interaction). For significant interactions, local treatment effects are marked out with asterisks

Deciduous shrub abundance and lichen cover were lower when large herbivores were present with no separate effect of small herbivores (T, Table 2; Figure 4). Herbivore presence influenced the abundance of evergreen shrubs, graminoids, and forbs, but this response differed among locations (T × L, Table 2; Figure 4). In S‐MH, evergreen shrubs were less abundant in the presence of herbivores, and a similar trend, although not significant, was seen in S‐DH. In the two Alaskan locations, abundance of evergreen shrubs did not differ across treatments (Figure 4). Graminoids were more abundant in the presence of large herbivores than in ungrazed controls in the dwarf shrub‐dominated S‐MH, while in the graminoid‐dominated A‐MAT, graminoid abundance was lower when small herbivores were present (Figure 4). Forbs were less abundant in A‐MAT when all herbivores were present compared to ungrazed controls, and both large and small herbivores seemed to contribute to this difference (Figure 4). For the three remaining locations, forbs were rare in all treatments and no differences were detected (Figure 4). In Alaska, standing dead graminoid abundance was lower in A‐MAT when herbivores were present which could be attributed to both small and large herbivores, while no exclosure effect was noticeable in A‐DH (T × L, Table 2; Figure 4).

**FIGURE 5:**
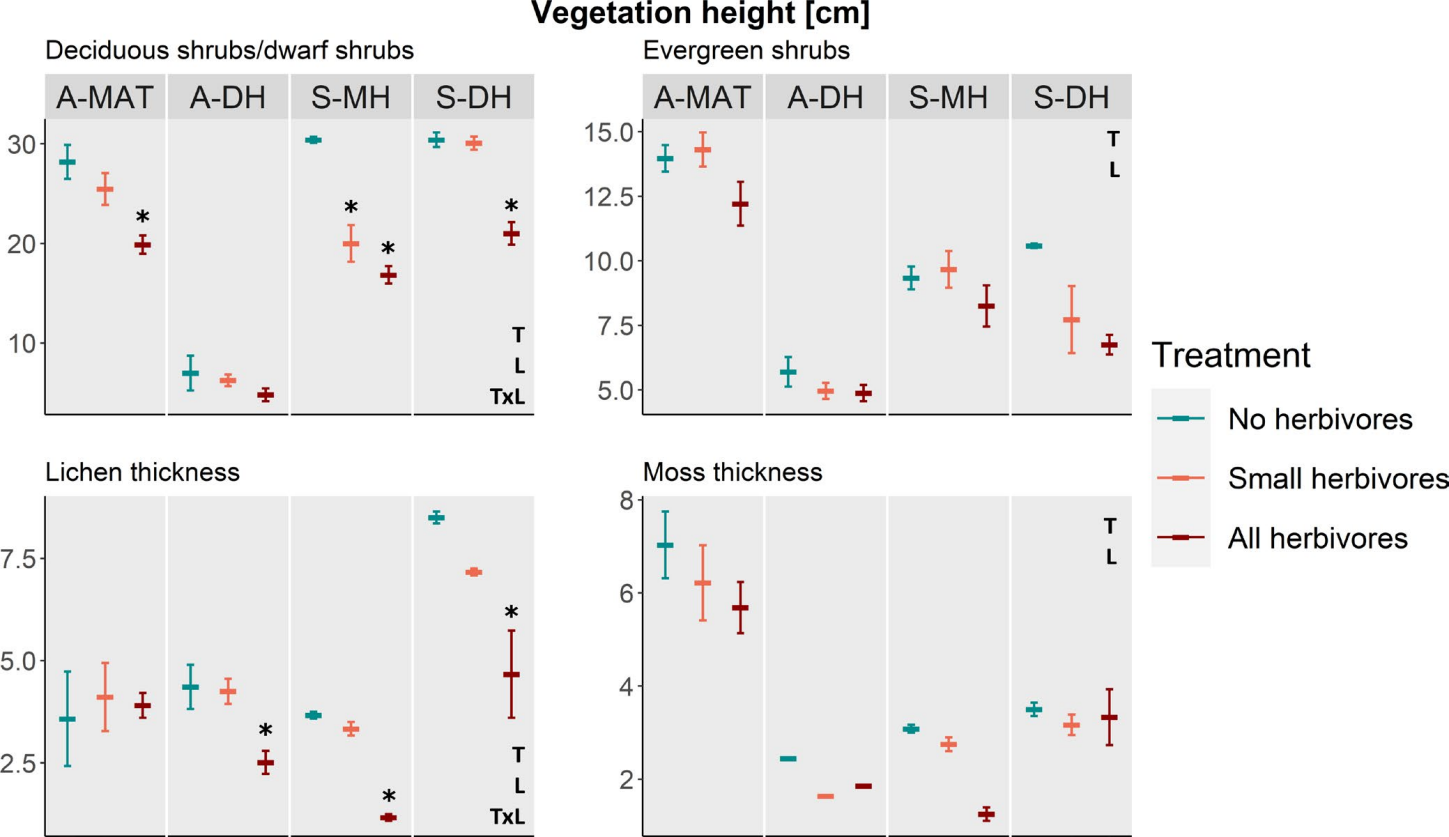
Effect of the presence of no herbivores (blue), small herbivores only (pink), and both small and large (all) mammal herbivores (red) on height of deciduous shrubs and dwarf shrubs, evergreen shrubs, and mat thickness of lichen and moss in four tundra vegetation types (A‐MAT = Alaskan moist acidic tundra, A‐DH = Alaskan dry heath, S‐MH = Scandinavian moist heath, S‐DH = Scandinavian dry heath). Plotted values are means ± SE and letters on the figure's right side indicate significant explanatory variables from linear mixed models (T = treatment, L = location, TxL = interaction). For significant interactions, local treatment effects are marked out with asterisks

The lichen layer was thinner with large herbivore presence in A‐DH, S‐MH, and S‐DH, while no herbivore effect was found in A‐MAT (T × L, Table 2; Figure 5). Deciduous shrub height was also lower with herbivores, but the effect differed among locations (T × L, Table 2; Figure 5). In A‐MAT and S‐DH, that effect was driven mainly by large herbivores, whereas in S‐MH, the small herbivores also had an effect. In A‐DH, no effect of herbivores on deciduous shrub height was found (Figure 5). The height of evergreen shrubs and the moss thickness were lower/thinner in the presence of all herbivores (T, Table 2; Figure 5), and no effect of small herbivores alone or difference in effect between locations could be statistically detected.

### 3.3 Species diversity

**FIGURE 6:**
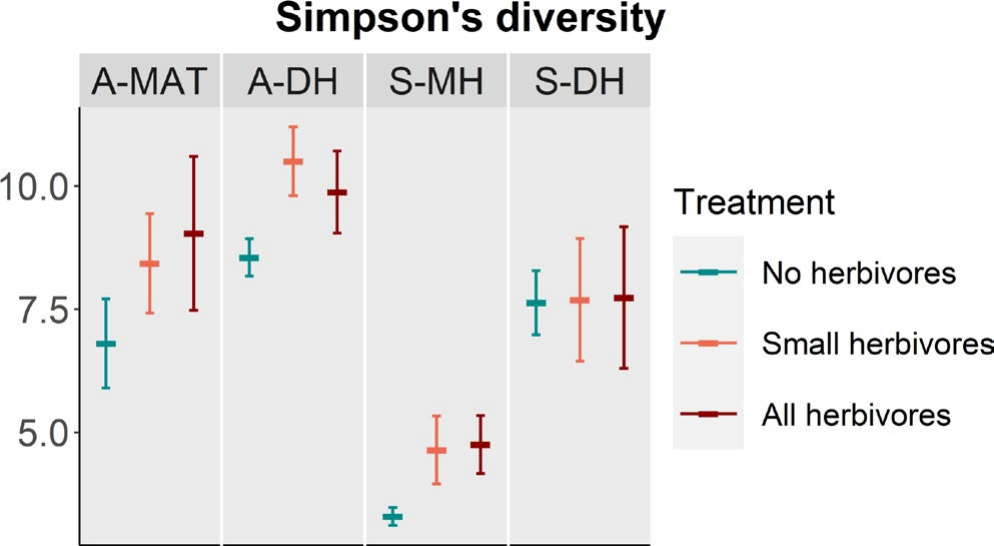
Effect of the presence of no herbivores (blue), small herbivores only (pink), and both small and large (all) mammal herbivores (red) on plant species diversity, measured as Simpson's diversity index, in four tundra vegetation types (A‐MAT = Alaskan moist acidic tundra, A‐DH = Alaskan dry heath, S‐MH = Scandinavian moist heath, S‐DH = Scandinavian dry heath). Plotted values are means ± SE and letters on the figure's right side indicate significant explanatory variables from linear mixed models (T = treatment, L = location, TxL = interaction)

Plant species diversity, measured as Simpson's diversity index, was on average higher whenever mammalian herbivores were present (T, Table 2; Figure 6). We saw no effects of herbivore presence in S‐DH, even though there was no statistical support for any differences in treatment effects among habitats (T × L, Table 2, Figure 6).

## 4 DISCUSSION

We investigated the importance of small and large herbivores on Arctic vegetation properties by studying long‐term exclosures in two contrasting vegetation types in both Scandinavia and Alaska. Herbivores had strong effects on the plant community composition and structure with possible implications on ecosystem functions on both continents and at all four study sites. However, which aspects of the plant community that were affected and what herbivore type caused the effect differed across sites. We found that the effects of herbivores on the plant communities varied more between contrasting habitats within continents than between continents, which partly supports our first hypothesis that small and large herbivores have similar effects on tundra vegetation in Scandinavia and Alaska. This further supports previous findings, as strong effects of both small and large herbivores have been reported across the Arctic (Berg et al., 2008; Bernes et al., 2015; Crête & Doucet, 1998; Gough et al., 2012; Manseau et al., 1996; Mulder & Harmsen, 1995; Olofsson et al., 2012; Pitelka & Batzli, 2007; Sundqvist et al., 2019; Zamin & Grogan, 2013). Although this study with only four locations cannot give a comprehensive description of how the importance of herbivory varies across the Arctic, it does indicate that differences in the role of small and large herbivores on ecosystem structure and function are shaped by habitat‐specific features rather than large biogeographical differences among continents.

The most apparent effect of herbivores was a lower density of field layer plants where (primarily small) herbivores were present. This effect was fairly strong as total number of plant hits were up to 50% lower when all mammalian herbivores were present in the productive habitats in both Alaska and Scandinavia, and NDVI had lower values with small herbivores present (−0.017) or all mammalian herbivores present (−0.020), compared to ungrazed controls. The latter might seem negligible, but is actually in the same range as the much discussed climate‐driven circumpolar increase in NDVI recorded by satellites during the last two decades (Xu et al., 2013; Zeng et al., 2017). This effect was only absent in A‐DH where field layer density was low in general and contained mainly prostrate dwarf shrubs that in general are less sensitive to grazing than more erect growth forms (Kaarlejärvi et al., 2017). The negative impact of herbivore presence on LAI and total plant hits in A‐DH has been associated with lower carbon uptake meaning that at least these dry heath tundra systems have the potential shift between being carbon sinks or sources depending on if herbivores are present or not (Min et al., 2021). In A‐MAT, S‐MH, and S‐DH, herbivory influenced all three measures related to plant density (LAI, NDVI, and total plant hits), but the strength of the effect differed among habitats and measurements. Herbivores reduced LAI and NDVI more in Scandinavia than in Alaska, while the effect on total plant hits was strongest in the most productive site in both continents. These three measures related to plant density provide information about different aspects of the ecosystem. For instance, in A‐MAT, the lower amount of total plant hits in the grazed plots was due to visually smaller and thinner tussocks of the dominant sedge *E. vaginatum* compared to tussocks in ungrazed controls (personal observation EL and JO). Earlier studies at the site have also observed both shorter leaves and less leaves per tiller in grazed tussocks (Gough et al., 2007). The lower number of hits when herbivores were present did not result in a lower NDVI or LAI, and several processes can be responsible for these contrasting responses between different measurement methods. It could be linked to saturation of NDVI in the productive habitats, increase of standing dead leaves reducing NDVI and compensating for the increase in green plants, and methodological challenges in measuring LAI inside dense turfs and in scarcely vegetated habitats.

In accordance with our second hypothesis, small mammalian herbivores had a stronger effect than large herbivores on many properties of the plant community, but some properties were more influenced by large herbivores. In all three habitats where herbivores decreased vegetation density, they also caused large differences in plant community composition, but the driving herbivore type differed among the three habitats. Small herbivores alone only had a substantial effect on plant community composition in A‐MAT, while both small and large herbivores were needed to affect the species composition in the two Scandinavian locations. We found the largest effects on species composition in the two moist habitats, while herbivores did not change the plant community in the driest site (A‐DH). These results correspond with the findings from a Finnish tundra (Saccone et al., 2014), where excluding all herbivores for three decades also resulted in contrasting vegetation communities, but more strongly so in moist compared to dry habitats.

To understand the herbivore effect on the plant communities better, the effect on separate plant functional groups needs to be considered. Probably the most commonly known effect of reindeer is that they reduce the abundance of ground lichens in summer and winter grazing ranges (Bernes et al., 2015; Gough et al., 2008; Olofsson et al., 2004; Roy et al., 2020). We did indeed find that large herbivores had a strong negative impact on lichen abundance in all places where ground lichens were common, but no clear effect of small herbivores. Another general effect of herbivores in arctic tundra is that they reduce the abundance of deciduous shrubs (Christie et al., 2015). This was found also in our study, and additionally, we found that both large and small herbivores contribute to this reduction. Small herbivores strongly reduced the abundance of evergreen shrubs in S‐MH, but had no clear effect at the other sites. In all sites, most evergreen shrubs were not preferred food for the herbivores, which could explain the small effect of herbivores on this functional group. This was in fact also the case in S‐MH, dominated by the evergreen and relatively unpalatable *E. hermaphroditum*, which is generally not eaten by herbivores (Tybirk et al., 2000). Here, the effect instead came from unselective disturbance by voles and lemmings, who cut *E. hermaphroditum* shoots to create runways and nests under the snow (Olofsson et al., 2012). In A‐MAT, graminoids was the functional group suffering the most from rodent impact. The dominant graminoid here was *E. vaginatum* which, in contrast to *E. hermaphroditum*, is preferred food by the common tundra vole, the most common rodent species in this community (Batzli & Lesieutre, 1995). Only a few signs of direct consumption were however observed in the field, suggesting that creation of burrows and runways during winter was probably a more important cause for this effect (Gough et al., 2012). In contrast to these negative effects, small herbivores had a positive effect on graminoids in S‐MH, allowing graminoids to establish in the empty space created by removal of the dominant evergreen shrubs. Standing dead plant material in Alaska followed the same pattern as graminoids, since almost all standing dead plants were dead *E. vaginatum* leaves within tussocks, and therefore closely connected to how much *E. vaginatum* was present.

Lower vascular plant height and thinner lichen and moss layers mostly came from addition of large herbivore activity. This can be explained by the fact that large herbivores graze the vegetation from above and therefore have the strongest impact on the tallest plants (Kaarlejärvi et al., 2017). Interestingly, the effects on plant height in our study were comparable to the effects expected from global warming. A study including 117 tundra warming experiments across the northern hemisphere (Bjorkman et al., 2018) found that an ubiquitous effect on plant communities is increased vegetation height and further predicted a 20%–60% increase at the end of this century. In our study, excluding herbivores for only 20–30 years lead to a similar increase in plant height for all growth forms (9%–44% percent) and especially deciduous shrubs (29%–44%). Together with other studies (Christie et al., 2015), our findings suggest that large herbivores have the capacity to dampen warming driven responses on plant height in tundra vegetation and thereby mitigate several negative climate change connected consequences on albedo (te Beest et al., 2016) and biodiversity (Kaarlejärvi et al., 2017).

In three out of four locations, we found strong positive effects on species diversity in plots with small herbivores. The effect seems to be independent of habitat productivity and thus contradicts the effect of large herbivores previously found in Scandinavia, where herbivores increased species diversity in productive sites, but decreased it in low‐productive sites (Sundqvist et al., 2019). In line with our third hypothesis, the positive effect we found on diversity relates to a lower abundance of competitively dominant plant species. In our study systems, small herbivores seem to increase species diversity by preventing dominance and create heterogeneity by creating burrows and runways (Olff & Ritchie, 1998). The spatial scale on which we recorded species diversity seem to catch local effects of rodents well (Hambäck et al., 1998), while the large‐scale effect from ungulate herbivores might be stronger at larger spatial scales.

Studies comparing a few locations cannot estimate how the effect of herbivores on vegetation in the tundra biome varies across the Arctic, but they can indicate which features of these interactions are general and which are site specific. One general effect in all our sites is that effects of herbivores are, at least partly, caused by disturbance (digging, trampling, and runways) rather than defoliation and actual consumption. This means that modeling the effect of herbivores based on their energy demands and food preferences will severely underestimate the actual effects of herbivores on the ecosystem (Yu et al., 2017). The impact of disturbance on different vegetation properties however differed among habitats. In SM‐H, rodents heavily affected NDVI by removing aboveground plant biomass when creating runways, while the effects in A‐MAT were more connected to rodents digging into tussocks to build burrows below the photosynthetically active part of the vegetation and therefore not detected by NDVI measurements.

To conclude, in this study, we surveyed the vegetation with the same method in the same year at four locations across the Arctic tundra. By doing so, we were able to detect general patterns and identify site specific interactions that could not be identified by meta‐analyses of already published data (Bernes et al., 2015), presumably because they were obscured by different surveying methods. Further studies using even more locations will be needed in the future to reveal how the strength of the interactions between herbivores and plants varies across the Arctic.

## CONFLICT OF INTEREST

The authors do not have any conflicts of interest to report.

## AUTHOR CONTRIBUTIONS

**Elin Lindén:** Conceptualization (lead); Data curation (lead); Formal analysis (lead); Funding acquisition (equal); Investigation (lead); Methodology (lead); Project administration (lead); Software (lead); Validation (lead); Visualization (lead); Writing‐original draft (lead); Writing‐review & editing (lead). **Laura Gough:** Funding acquisition (equal); Resources (equal); Supervision (equal); Writing‐review & editing (equal). **Johan Olofsson:** Conceptualization (equal); Funding acquisition (lead); Methodology (equal); Supervision (lead); Writing‐review & editing (equal).

## Supporting information

Figure S1‐S3

## Data Availability

Data will be deposited in the Dryad Digital repository https://doi.org/10.5061/dryad.rfj6q57b6.
