# Supplementary material for: Large and small herbivores have strong effects on tundra vegetation in Scandinavia and Alaska
Source: Ecol Evol. 2021 Aug 2;11(17):12141–52. doi: 10.1002/ece3.7977 (PMC8427618; doi:10.1002/ece3.7977)
Supplement: Supplementary file 1 — Figure S1‐S3 [file ECE3-11-12141-s001.docx]

**Supporting information**

Figure S1.

Fig S1. Monthly precipitation and mean temperature during 2018, the year of inventory, in two scandinavian tundra sites in Abisko and Joatka and two alaskan sites geographically, close to each other, in Toolik lake. Data retrieved from Environmental Data Center, Toolik Alaska, Abisko research station and Norske Meteorologiske Institut.

Figure S2.


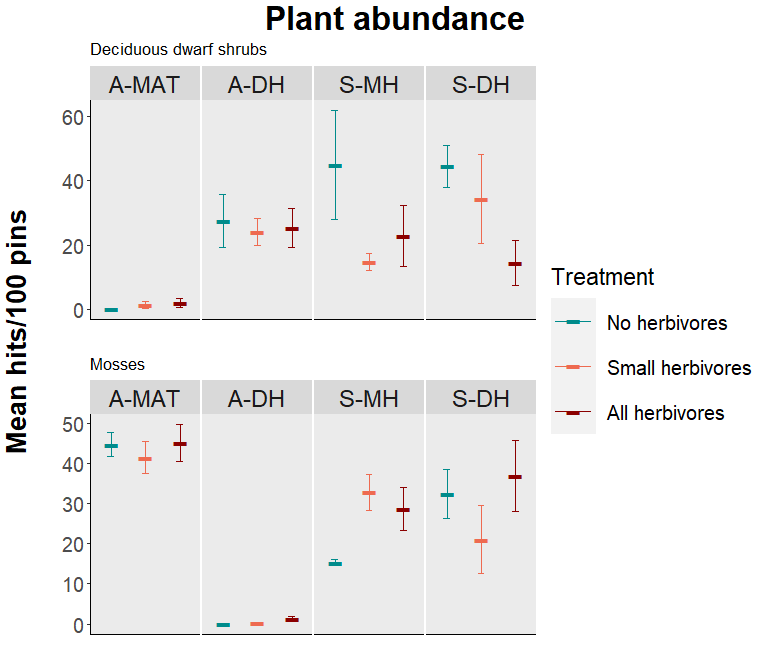


Fig S2. Effect of the presence of no herbivores (blue), small herbivores only (pink) and both small and large (all) mammal herbivores (red) on the abundance of deciduous dwarf shrubs and mosses in four tundra locations (A-MAT=Alaskan Moist Acidic Tundra, A-DH=Alaskan Dry Heath, S-MH=Scandinavian Moist Heath, S-DH=Scandinavian Dry Heath). Plotted values are means±SE.

Figure S3


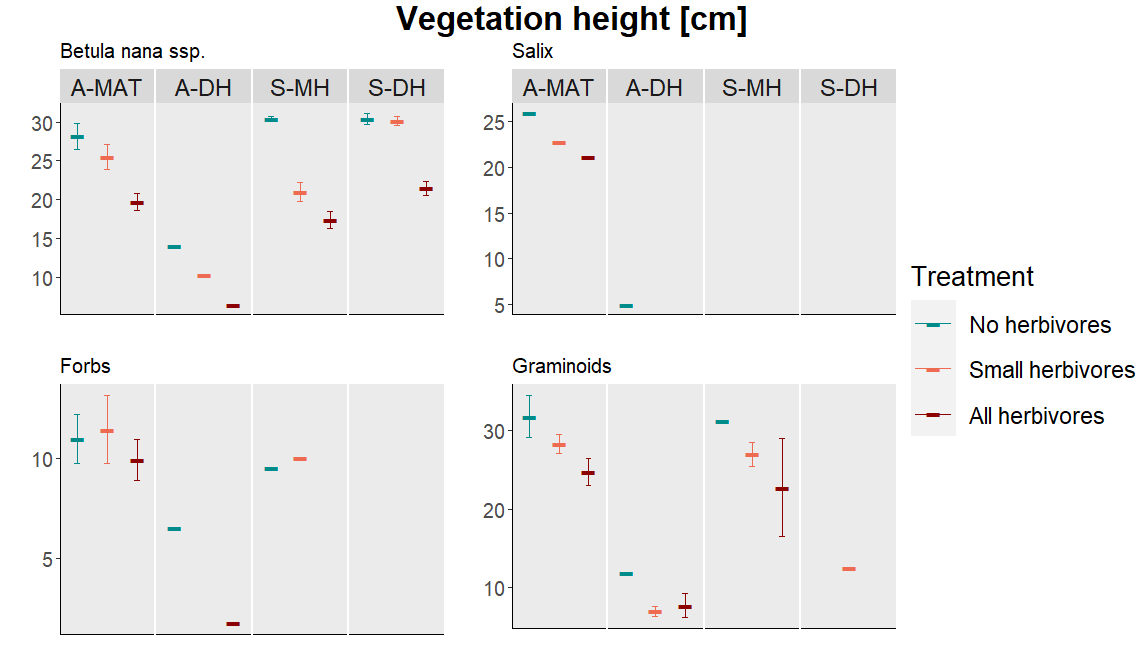


Fig S3. Effect of the presence of no herbivores (blue), small herbivores only (pink) and both small and large (all) mammal herbivores (red) on height of *Betula nana* ssp., *Salix* sp., forbs and graminoids in four tundra vegetation types (A-MAT = Alaskan Moist Acidic Tundra, A-DH = Alaskan Dry Heath, S-MH=Scandinavian Moist Heath, S-DH=Scandinavian Dry Heath). Plotted values are means±SE.
